# Silencing RNA for MMPs May Be Utilized for Cardioprotection

**DOI:** 10.1155/2022/9729018

**Published:** 2022-08-24

**Authors:** Marta Banaszkiewicz, Anna Krzywonos-Zawadzka, Agnieszka Olejnik, Agnieszka Noszczyk-Nowak, Iwona Bil-Lula

**Affiliations:** ^1^Division of Clinical Chemistry and Laboratory Hematology, Department of Medical Laboratory Diagnostics, Faculty of Pharmacy, Wroclaw Medical University, Borowska 211 A, 50-556 Wroclaw, Poland; ^2^Department of Internal Medicine and Clinic of Diseases of Horses, Dogs and Cats Faculty of Veterinary Medicine, Wroclaw University of Environmental and Life Sciences, pl. Grunwaldzki 47, 50-366 Wroclaw, Poland

## Abstract

Ischemia/reperfusion (I/R) injury is accompanied by an increase of matrix metalloproteinase 2 (MMP-2) activity, which degrades heart contractile proteins. The aim of the study was to investigate the effect of MMP-2 small interfering RNA (MMP-2 siRNA) administration on I/R heart. Isolated rat hearts perfused by the Langendorff method were subjected to I/R in the presence or absence of MMP-2 siRNA. The hemodynamic parameters of heart function were monitored. Lactate dehydrogenase (LDH) activity was measured in coronary effluents. Activity and concentration of MMPs in the hearts were measured. Concentration of troponin I (TnI) in coronary effluents was examined as a target for MMP-2 degradation. Recovery of heart mechanical function was reduced after I/R; however, administration of MMP-2 siRNA resulted in restoration of proper mechanical function (*p* < 0.001). LDH activity was decreased after the use of MMP-2 siRNA (*p* = 0.02), providing evidence for reduced cardiac damage. Both MMP-2 and MMP-9 syntheses as well as their activity were inhibited in the I/R hearts after siRNA administration (*p* < 0.05). MMP-2 siRNA administration inhibited TnI release into the coronary effluents (*p* < 0.001). The use of MMP-2 siRNA contributed to the improvement of heart mechanical function and reduction of contractile proteins degradation during I/R; therefore, MMP-2 siRNA may be considered a cardioprotective agent.

## 1. Introduction

Matrix metalloproteinases (MMPs) are proteolytic enzymes whose main physiological role is to maintain the structural integrity of the extracellular matrix (ECM) [1–4]. However, there are studies showing that matrix metalloproteinase 2 (MMP-2), located in most types of heart cells including endothelial cells, vessels, smooth muscle cells, fibroblasts, and cardiomyocytes, has also an intracellular function—it contributes to the degradation of contractile proteins, such as myosin light chain 1 (MLC1), troponin I (TnI), or titin [5–7], leading to weakening of cardiomyocytes contractility [8]. This is of particular importance in ischemia/reperfusion (I/R) heart injury, when the synthesis and activity of MMP-2 are intensified due to oxidative stress within seconds of ischemia [9–12]. Therefore, the mechanical properties of the heart tissue are reduced, leading to a deterioration of heart function [13]. The injury of heart tissue primarily happens in the first minutes of reperfusion, when reactive oxygen species (ROS) are synthesized [14]. The degradation of contractile proteins is considered to be the main cause of heart damage, but it has also been shown that systolic dysfunction may be a reversible process [8, 15]. Under physiological conditions, MMPs are inhibited by specific endogenous tissue inhibitors of metalloproteinases (TIMPs) [16–18]. TIMP-4 dominates in MMP inhibition in the heart; however, it is exclusively released from cardiomyocytes during the infarction; hence, a further increase in MMP-2 activity during reperfusion is observed [15, 19, 20]. For this reason, the reduction of MMP-2 activity has become an important therapeutic target [13]. Since small interfering RNA (siRNA) for MMP-2 was created, it is possible to reduce the expression of *MMP-2* gene [8, 21, 22]. siRNA mutes the target gene by inhibiting the translation of a single, complementary messenger RNA (mRNA), which in turn should affect the protein activity. MMP-2 siRNA should act specifically on MMP-2 mRNA and possibly on other MMPs with corresponding mRNA sequence, while TIMPs have a broad inhibitory effect on many MMPs [1, 9, 21, 23].

The use of MMP-2 siRNA may be of great clinical importance in preventing damage caused by increased oxidative stress due to I/R and deterioration of contractile function associated with the degradation of contractile proteins. Thus, the main aim of the study was to investigate the effect of MMP-2 siRNA administration on heart subjected to ischemia/reperfusion injury. We explored that MMP-2 siRNA may constitute a cardioprotective factor during I/R injury of the heart as its administration to heart inhibited contractile proteins degradation, heart damage, and restored proper heart mechanical functions.

## 2. Materials and Methods

This investigation conforms to the Guide to the Care and Use of Experimental Animals published by the Polish Ministry of Science and Higher Education and was approved by the local Ethics Committee for Experiments on Animals at the Ludwik Hirszfeld Institute of Immunology and Experimental Therapy, Polish Academy of Sciences, Wroclaw, Poland (resolution 085/2020/P1 of 9^th^ December 2020 and 034/2022 of 20^th^ July 2022).

### 2.1. Heart Perfusion with Langendorff Method

**Figure 1:**
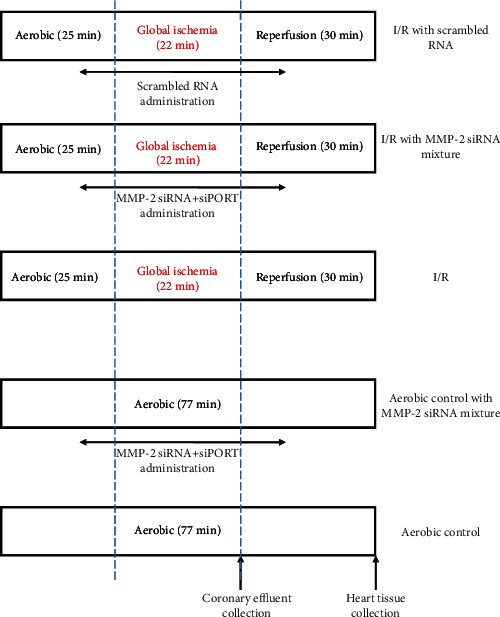
Experimental protocol for I/R and aerobic control with or without MMP-2 siRNA/scrambled RNA and siPORT mixture infusion. The hearts were perfused with MMP-2 siRNA/scrambled RNA and siPORT mixture for the last 10 minutes of aerobic stabilization and the first 10 minutes of reperfusion. I/R: ischemia-reperfusion.

10- to 11-week old male Wistar rats (weighing 300-350 g) were used in these experiments as a surrogate model for analysis of cardioprotection [24]. Rats were treated with buprenorfin (0.05 mg/kg, i.p.) and anesthetized with sodium pentobarbital (40 mg/kg, i.p.). The hearts were rapidly excised from animals and rinsed by immersion in ice-cold Krebs-Henseleit Buffer containing 118 mmol/L NaCl, 4.7 mmol/L KCl, 1.2 mmol/L KH_2_PO_4_, 1.2 mmol/L MgSO_4_, 3.0 mmol/L CaCl_2_, 25 mmol/L NaHCO_3_, 11 mmol/L glucose, and 0.5 mmol/L EDTA, pH 7.4. Immediately after removal, spontaneously beating hearts were suspended on a blunt end needle of the Langendorff system (EMKA Technologies, Paris, France) with aorta and maintained at 37°C. Hearts were perfused at a constant pressure of 60 mmHg with Krebs-Henseleit Buffer at pH 7.4, at 37°C and gassed continuously with 5% CO_2_/95% O_2_. After stabilization, the hearts were subjected to global—no flow I/R injury (Figure 1). The temperature of the heart during ischemia was maintained by closing the EMKA system chamber, where the heart was located—the chamber was connected to a water bath forcing a liquid at 37°C. Coronary flow (CF), heart rate (HR), and left ventricular developed pressure (LVDP) were defined as hemodynamic end-points of cardioprotection and monitored using an EMKA recording system with IOX2 software (EMKA Technologies, Paris, France) [25]. A water-filled latex balloon, which was connected to the pressure transducer and inserted through an incision in the left atrium into the left ventricle through the mitral valve, measured HR and LVDP. The volume of the buffer was adjusted at the beginning of the perfusion to achieve an end-diastolic pressure 8-10 mmHg. LVDP was calculated as the difference between peak systolic and diastolic pressures. Cardiac mechanical function was expressed as the product of HR and LVDP (systolic minus diastolic ventricular pressures)—rate pressure product at 77 minutes versus 25 minutes of perfusion (RPP). At the end of protocol, hearts were immediately submerged in liquid nitrogen and stored at -80°C before further investigations.

### 2.2. siRNA Transfection

A mixture containing a pool of 3 target-specific 19-25 nucleotide small interfering RNAs (MMP-2 siRNA) designed to reduce expression of rat *MMP-2* gene (Santa Cruz Biotechnology, Dallas, Texas, United States) was resuspended in siRNA Dilution Buffer (Santa Cruz Biotechnology, Dallas, Texas, United States) to a final 10 *μ*M solution in a 10 *μ*M Tris-HCl, 20 mM NaCl, and 1 mM EDTA buffered solution at pH 8.0. This solution was stored at -20°C. To transfect siRNAs into heart myocytes, siPORT Amine Polyamine-Based Transfection Agent (Invitrogen, Carlsbad, California, United States) was used. siPORT amine polyamine-based tranfection agent is a proprietary blend of polyamins formulated for transfection of small siRNAs into cells. The reagent works by complexing with RNA and facilitating its transfer into cells. siPORT amine agent was diluted in ice-cold Krebs-Henseleit Buffer (containing 118 mmol/L NaCl, 4.7 mmol/L KCl, 1.2 mmol/L KH_2_PO_4_, 1.2 mmol/L MgSO_4_, 3.0 mmol/L CaCl_2_, 25 mmol/L NaHCO_3_, 11 mmol/L glucose, and 0.5 mmol/L EDTA, pH 7.4) in 1 : 9 ratio (*ν* : *ν*). Subsequently, 330 pmol siRNA was mixed with siPORT amine agent in equal volume and infused into hearts in 250 mL of Krebs-Henseleit buffer (at 37°C). Scrambled RNA (scr RNA, control siRNA-A, Santa Cruz Biotechnology, Dallas, Texas, United States), consisting of scrambled sequence that will not lead to the specific degradation of any cellular message, was prepared and treated the same as MMP-2 siRNA.

### 2.3. Global Ischemia/Reperfusion of Isolated Rat Hearts

After 25 minutes of aerobic stabilization, the hearts were subjected to 22 minutes of global, no flow ischemia [26]. Then, hearts were perfused in aerobic conditions for 30 minutes (reperfusion). In the study group, following 15 minutes of aerobic perfusion, a mixture of MMP-2 siRNA/scrambled RNA (330 pmol) and siPORT amine agent was infused for 10 minutes in Krebs-Henseleit Buffer into the aerobically perfused heart and for 10 minutes at the beginning of reperfusion—standard Krebs-Henseleit Buffer was replaced with 250 mL of Krebs-Henseleit Buffer with mixture of siRNA/scrRNA and siPORT (Figure 1). Mechanical function of the heart (RPP) was evaluated at the end of aerobic perfusion (25 minutes) and at the end of reperfusion (77 minutes). Coronary effluents for biochemical tests were collected at the beginning of reperfusion (47 minutes) to achieve the constant volume (15 mL).

### 2.4. Preparation of Heart Homogenates

The hearts previously frozen at -80°C were crushed in liquid nitrogen using a mortar and pestle. Then, the hearts underwent three cycles of freezing in liquid nitrogen and thawing at 37°C in the homogenization buffer containing 50 mmol/L Tris-HCl (pH 7.4), 3.1 mmol/L sucrose, 1 mmol/L dithiothreitol, 10 mg/mL leupeptin, 10 mg/mL soybean trypsin inhibitor, 2 mg/mL aprotinin, and 0.1% Triton X-100. The homogenates were centrifuged at 10 000 x g at 4°C for 15 minutes. Supernatants were collected and stored at -80°C.

### 2.5. Protein Concentration Testing

The Bradford method was used to determine total protein concentration in cardiac tissue homogenates. Bovine serum albumin (BSA, heat shock fraction, ≥98%, Sigma-Aldrich, Saint Louis, Missouri, United States) served as the protein standard. Bio-Rad Protein Assay Dye Reagent (Bio-Rad, Hercules, California, United States) and Spark multimode microplate reader (Tecan Trading AG, Mannedorf, Switzerland) were used for measuring total protein concentration.

### 2.6. Assessment of LDH Activity

To determine the activity of LDH in coronary effluents, Lactate Dehydrogenase Activity Assay Kit (Sigma-Aldrich, Saint Louis, Missouri, United States) was used according to manufacturer's instruction. LDH is a stable cytosolic enzyme that serves as a marker of cell damage because it is released upon membrane damage/permeability or cell lysis. LDH level in I/R/I/R+scrRNA and I/R+MMP-2 siRNA coronary effluents was normalized to CF and compared.

### 2.7. MMP-2 and MMP-9 mRNA Expressions in Heart Tissue

TRIzol reagent (ThermoFisher Scientific, Waltham, MA, USA) was used to isolate total RNA from the heart tissue according to the manufacturer's instruction. Reverse transcription of pure RNA (2 *μ*g) was performed with the use of iScript cDNA Synthesis Kit (Bio-Rad, Hercules, CA, USA). The expression of *MMP-2/MMP-9* gene in relation to glyceraldehyde 3-phosphate dehydrogenase (*GAPDH*) gene was analyzed by relative RQ-PCR using CFX96 Touch (Bio-Rad, Hercules, CA, USA). iTaq Universal Sybr Green Supermix (Bio-Rad, Hercules, CA, USA), forward and reverse primers (final concentration 0.1 *μ*M/L), Ultra Pure DEPC-treated water (Thermo Fisher Scientific, Waltham, MA, USA), and cDNA (100 ng) were used in a final volume of 30 *μ*L. The 5-3′ sequences of primers were as follows: *MMP-2* F: AGCAAGTAGACGCTGCCTTT, *MMP-2* R: CAGCACCTTTCTTTGGGCAC; *MMP-9* F: GATCCCCAGAGCGTTACTCG, *MMP-9* R: GTTGTGGAAACTCACACGCC; and *GAPDH* F: AGTGCCAGCCTCGTCTCATA, *GAPDH* R: GATGGTGATGGGTTTCCCGT. The amount of mRNA in relation to *GAPDH* was calculated as 2^−*Δ*Ct^. The relative expression of *MMP-2/MMP-9* gene was compared in hearts exposed to aerobic conditions (control group) and in hearts subjected to global ischemia with/without siRNA/scrRNA administration.

### 2.8. MMP-2 and MMP-9 Protein Levels in Heart Homogenates

MMP-2 and MMP-9 concentrations in heart homogenates were measured quantitatively using Quantikine ELISA Assay for Total MMP-2 (R&D Systems, Minneapolis, Minnesota, United States) and rat total MMP-9 Quantikine ELISA Kit (R&D Systems, Minneapolis, Minnesota, United States) according to the manufacturer's instruction. Total MMP-2 Quantikine ELISA Assay recognized recombinant MMP-2, natural human, mouse, rat, porcine, and canine active, pro-, and TIMP-complexed MMP-2, while Rat Total MMP-9 Quantikine ELISA Kit measured total rat MMP-9 (pro-, active, and TIMP-complexed MMP-9). MMP-2/MMP-9 immobilized with monoclonal antibody specific to appropriate proteins were detected using anti-MMP-2 or anti-MMP-9 polyclonal antibody conjugated to horse-radish peroxidase (HRP). TMB substrate solution was used to develop the reaction. A minimum detectable dose was 0.033 and 0.013 ng/mL, respectively. MMP-2/MMP-9 concentration in heart homogenates was expressed as ng per *μ*g of total protein.

### 2.9. Gelatin Zymography

Gelatin zymography for measurement of MMP-2 and MMP-9 activities in heart homogenates was performed with Heussen and Dowdle protocol modified by us [27, 28]. Heart homogenates were adjusted to the same protein concentration and were mixed with 4x Laemmli Sample Buffer (Bio-Rad Laboratories, Hercules, California, United States) in 4 : 1 ratio (*ν* : *ν*). Samples containing 80 *μ*g of protein were applied to 8% polyacrylamide gel copolymerized with gelatin (2 mg/mL) and 0.1% SDS (denaturing, but not reducing conditions). After electrophoresis, gels were rinsed in 2.5% Triton X-100 (3 times for 20 minutes) to remove SDS. Then, gels were placed in the incubation buffer (50 mol/L Tris-HCl pH 7.5, 5 mmol/L CaCl_2_, 200 mmol/L NaCl, and 0.05% NaN_3_) at 37°C overnight. After digestion of gelatin by enzymes, gels were stained in staining solution (0.5% Coomassie Brilliant Blue R-250, 30% methanol, 10% acetic acid) for 2 h and destained in destaining solution (30% methanol, 10% acetic acid) until bands were clearly visible. MMP-2 and MMP-9 activities were visualized as bright bands on a dark background. HT10-80 cell line and PRP were used as positive controls. Zymograms were scanned using GS-800 Calibrated Densitometer (model PowerLook 2100 XL-USB) and analyzed using Quantity One v. 4.6.9 software (Bio-Rad Laboratories, Hercules, California, United States). The relative activity of MMP-2/MMP-9 was established and expressed in arbitrary units (AU) as activity per *μ*g of total protein.

### 2.10. TIMP-4 Level in Heart Homogenates

The metalloproteinase tissue inhibitor 4 (TIMP-4) level in heart homogenates was measured using ELISA Kit for Rat Tissue Inhibitors of Metalloproteinase 4 (USCN Life Science Inc., Wuhan, China). Briefly, capture antibody bound TIMP-4 from heart homogenates. Specific biotin-conjugated polyclonal antibody detected the antigen and next was conjugated with HRP. TMB substrate solution was added to develop the reaction. The minimum detectable dose of rat TIMP-4 was less than 0.041 ng/mL. TIMP-4 concentration in heart homogenates was expressed as ng per *μ*g of total protein.

### 2.11. MLC1 Concentration in Coronary Effluents

Myosin light chain 1 (MLC1) concentration in coronary effluents was determined with the use of Rat Myosin Light Chain 3 ELISA Kit (sensitivity: 6.9 × 10^−3^ ng/mL; Bioassay Technology Laboratory, Birmingham, United Kingdom). Briefly, capture antibody bound MLC1 from coronary effluents. Next, antigens were detected by biotinylated antibody. HRP conjugated with biotinylated MLC1 antibody. TMB substrate solution was used to develop the reaction. MLC1 concentration in coronary effluents was expressed as ng/L and normalized to CF.

### 2.12. TnI Concentration in Coronary Effluents

Cardiac troponin I was quantitatively measured with the use of Rat Cardiac Troponin I SimpleStep ELISA Kit (Abcam, Cambridge, United Kingdom) in coronary effluents. Troponin I was tied with antibody specific to rat cardiac muscle troponin I and was detected by biotin-conjugated polyclonal antibody and HRP. Next, TMB Development Solution was used to enable visualization of the reaction. A minimum detectable dose of rat cardiac TnI was 7.7 pg/mL. TnI concentration in coronary effluents was expressed as pg/mL and normalized to CF.

### 2.13. Statistical Analysis

Experimental data were analyzed using GraphPad Prism 6 software (GraphPad Software). Shapiro-Wilk normality test or Kolmogorov-Smirnov test was used to assess normality of variances changes. Student's *t* test or Mann–Whitney *U* test was used for comparison between two groups. One-way ANOVA with Tukey's test as the post hoc test or Kruskal-Wallis and Dunn's test for multiple groups were used. Correlations were assessed using Pearson's or Spearman's test, as appropriate. Results were expressed as mean ± SEM. *p* < 0.05 was the criterion for statistical significance. *N* number in all tests was 4-8.

## 3. Results

### 3.1. MMP-2 siRNA Affects MMP-2 and MMP-9 Gene Expressions, Proteins Synthesis, and Activity in Cardiac Tissue

**Figure 2:**
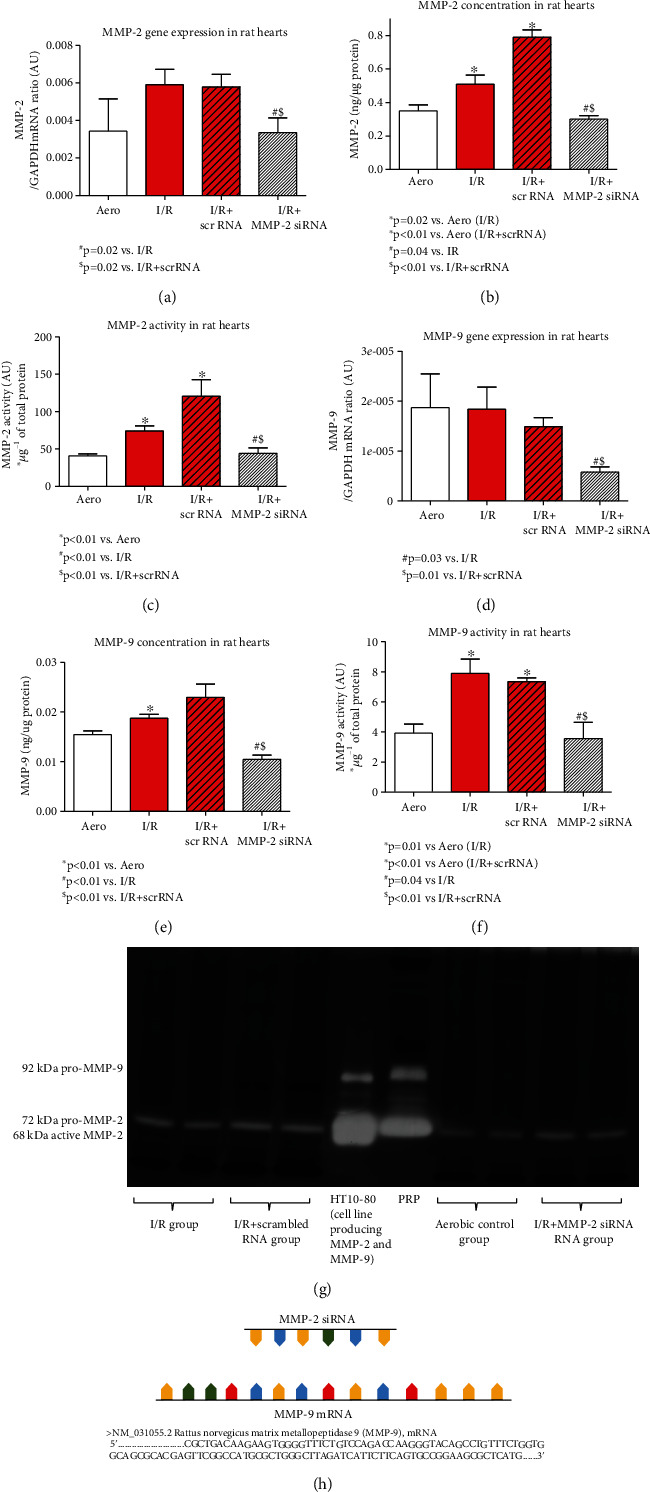
*MMP-2* gene expression (a), concentration (b), and activity (c) in heart homogenates and *MMP-9* gene expression (d), concentration (e), and activity (f) in the heart tissue. Representative zymogram of MMP-2/MMP-9 activity in heart homogenates (HT10-80 and PRP were used as standards for pro-/active MMP-2 and MMP-9) (g). Graph presenting the site of siRNA binding to the fragment of MMP-9 mRNA sequence (h). Aero: aerobic control group; I/R: ischemia/reperfusion; MMP-2: matrix metalloproteinase 2; MMP-9: matrix metalloproteinase 9; PRP: platelet-rich plasma.

To explore changes in *MMP-2* gene expression, protein synthesis, and activity under the influence of MMP-2 siRNA, we measured *MMP-2* gene expression using RQ-PCR. We also assessed MMP-2 concentration using ELISA test and performed gelatin zymography. Synthesis and activity of MMP-2 were significantly increased in rat hearts subjected to I/R (without and with scrRNA (I/R: *p* = 0.02, *p* < 0.01; I/R+scrRNA: *p* < 0.01, *p* < 0.01 vs. the Aero group, respectively), showing oxidative stress activation of matrix metalloproteinase. The use of MMP-2 siRNA during I/R inhibited *MMP-2* gene expression, synthesis, and activity (*p* = 0.02, *p* = 0.04, *p* < 0.01 vs. the I/R group; *p* = 0.02, *p* < 0.01, and *p* < 0.01 vs. the I/R+scrRNA group, respectively) (Figures 2(a)–2(c) and 2(g)). Since MMP-2 and MMP-9 present conserved sequence in central part of the gene, we checked that the siRNA sequence used in this experiment could also attach to the fragment of MMP-9 mRNA sequence (Figure 2(h)); hence, we revealed that MMP-9 synthesis and activity changed in the same manner as MMP-2 parameters—they were substantially increased during I/R (I/R: *p* < 0.01, *p* = 0.01; I/R+scrRNA: *p* = NS, *p* < 0.01 vs. Aero, respectively), and administration of siRNA resulted in inhibited gene expression, synthesis, and activity of MMP-9 (*p* = 0.03, *p* < 0.01, and *p* = 0.04 vs. I/R; *p* = 0.01, *p* < 0.01, and *p* < 0.01 vs. the I/R+scrRNA group, respectively) (Figures 2(d)–2(f)).

### 3.2. Effect of MMP-2 siRNA on Mechanical Function, Coronary Blood Flow, and Heart Injury

**Figure 3:**
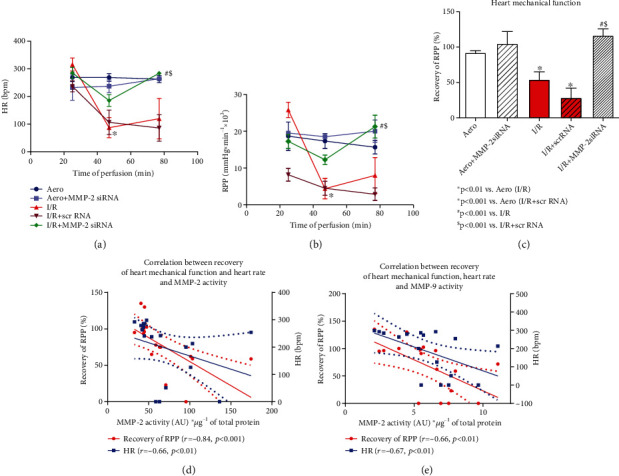
An influence of MMP-2 siRNA on heart function: heart rate (a). RPP calculated as the product of the heart rate and left ventricular developed pressure (heart rate x left ventricular developed pressure/1000) (b). Recovery of heart mechanical function (difference between RPP at 25^th^ and 77^th^ min of perfusion expressed as a percentage of RPP recovery) (c). Correlation between heart mechanical function recovery, heart rate and MMP-2 activity (d); MMP-9 activity (e) in heart homogenates; straight line: standard curve; dotted line: 95% confidence band. Aero: aerobic control group; bpm: beats per minute; HR: heart rate; I/R: ischemia-reperfusion group; MMP-2: matrix metalloproteinase 2; MMP-9: matrix metalloproteinase 9; RPP: rate pressure product. Mean ± SEM, *n*_Aero_ = 3; *n*_Aero+MMP−2 siRNA_ = 2; *n*_I/R_ = 5; *n*_I/R+scrRNA_ = 5; *n*_I/R+MMP−2 siRNA_ = 4. ^∗^*p* < 0.05 vs. Aero, ^#^*p* < 0.05 vs. I/R, and ^$^*p* < 0.05 vs. I/R+scrRNA.

Cardiac hemodynamic parameters were significantly decreased after the I/R protocol (Table 1, Figures 3(a)–3(c)). Recovery of heart mechanical function was decreased approximately by 40% in I/R hearts compared to aerobically perfused organs (approximately 93% recovery) (Figure 3(c)). To check if MMP-2 siRNA was not toxic to the hearts, we perfused hearts in aerobic conditions with the administration of MMP-2 siRNA—the hemodynamic parameters were not significantly different from the aerobic control (Table 1, Figures 3(a)–3(c)). Perfusion of hearts subjected to I/R with MMP-2 siRNA resulted in increased recovery of the heart mechanical function (*p* < 0.001), as well as heart rate (HR) (*p* < 0.01), left ventricular developed pressure (LVDP) (*p* = 0.01), and coronary flow (CF) (*p* < 0.01) (Table 1, Figure 3). Moreover, we revealed that recovery of the heart mechanical function as well as heart rate negatively correlated with MMP-2 (*p* < 0.001, *r* = −0.84 and *p* < 0.01, *r* = −0.66, respectively) and MMP-9 activity (*p* < 0.01, *r* = −0.66 and *p* < 0.01, *r* = −0.67), confirming the role of MMP-2 and MMP-9 in heart function disturbances (Figures 3(d) and 3(e)).

**Figure 4:**
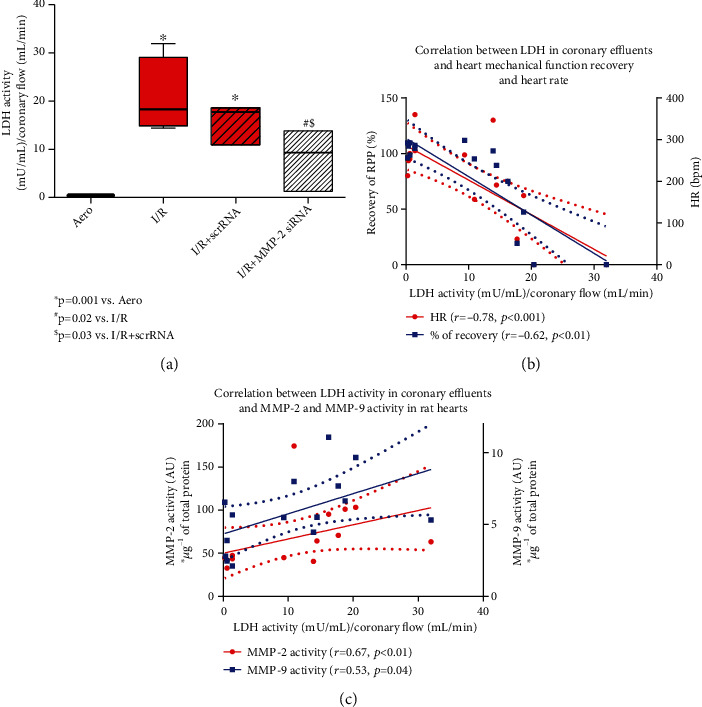
LDH activity in coronary effluents (a). Correlation between LDH activity in coronary effluents and recovery of heart mechanical function and heart rate (b) and correlation between LDH activity in coronary effluents and MMP-2 and MMP-9 activity in heart homogenates (c). Straight line: standard curve; dotted line: 95% confidence band. Aero: aerobic control group; I/R: ischemia/reperfusion; HR: heart rate; LDH: lactate dehydrogenase; MMP-2: matrix metalloproteinase 2; MMP-9: matrix metalloproteinase 9.

Lactate dehydrogenase (LDH) activity, used as a marker of cellular injury, was measured in coronary effluents. LDH activity was significantly increased in the I/R and I/R+scrRNA groups (*p* = 0.001 vs. Aero), confirming cell damage. Administration of MMP-2 siRNA into hearts subjected to I/R reduced LDH activity in coronary effluents (*p* = 0.02 vs. I/R, *p* = 0.03 vs. I/R+scrRNA), pointing to cardioprotection (Figure 4(a)). Moreover, strong negative correlation between LDH and recovery of heart contractile function (*r* = −0.62, *p* < 0.01) as well as HR (*r* = −0.78, *p* < 0.001) was observed (Figure 4(b)), showing heart dysfunction due to I/R injury. Strong positive correlation of LDH activity and MMP-2 and MMP-9 activities (*p* < 0.01, *r* = 0.67; *p* = 0.04, *r* = 0.53, respectively) indicated the role of MMPs in heart damage (Figure 4(c)).

### 3.3. Influence of MMP-2 siRNA on Degradation of Heart Contractile Proteins

**Figure 5:**
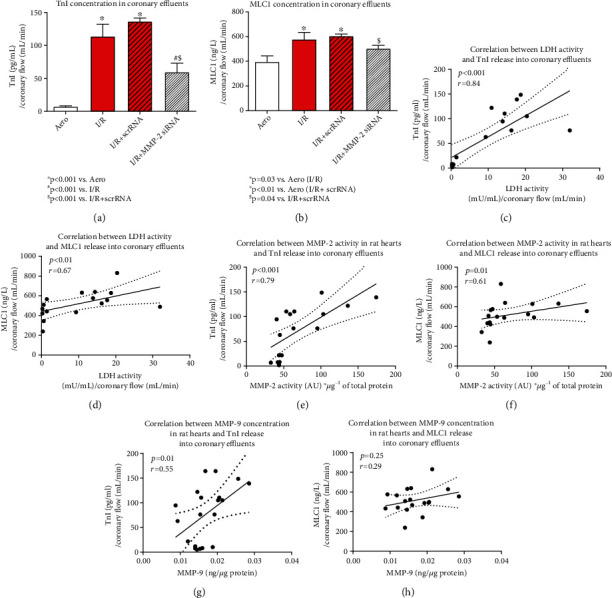
TnI (a) and MLC1 (b) release into coronary effluents. Correlation between LDH activity and TnI (c) and MLC1 (d) release. Correlation between MMP-2 activity and TnI (e) and MLC1 (f) release into extracellular space. Correlation between MMP-9 synthesis in rat hearts and TnI (g) and MLC1 (h) release into coronary effluents. Straight line: standard curve; dotted line: 95% confidence band. Aero: aerobic control group; I/R: ischemia/reperfusion; LDH: lactate dehydrogenase; MLC1: myosin light chain 1; MMP-2: matrix metalloproteinase 2; TnI: troponin I.

To explore the influence of MMPs mRNA silencing on heart contractile apparatus, we measured troponin I (TnI) and myosin light chain 1 (MLC1) levels in effluents. We showed that I/R caused increased release of both TnI and MLC1 into the effluents (I/R: *p* < 0.001, *p* = 0.03; I/R+scrRNA: *p* < 0.001, *p* < 0.01 vs. Aero, respectively), but siRNA administration resulted in decreased TnI (*p* < 0.001 vs. I/R, *p* < 0.001 vs. I/R+scrRNA) as well as MLC1 release (*p* = NS vs. I/R, *p* = 0.04 vs. I/R+scrRNA) (Figures 5(a) and 5(b)). Both TnI and MLC1 concentrations in coronary effluents positively correlated with LDH activity (*p* < 0.001, *r* = 0.84; *p* < 0.01, *r* = 0.67, respectively) (Figures 5(c) and 5(d)) and MMP-2 activity in rat hearts (*p* < 0.001, *r* = 0.79; *p* = 0.01, *r* = 0.61, respectively) (Figures 5(e) and 5(f)) and a positive correlation between TnI release and MMP-9 synthesis in the heart tissue (*p* = 0.01, *r* = 0.55) (Figure 4(g)) was also observed, confirming the degradation of contractile proteins due to increased activity of MMPs. MLC1 concentration in coronary effluents did not correlate with MMP-9 synthesis (Figure 5(h)).

### 3.4. MMP-2 siRNA Affects TIMP-4 in Hearts during I/R

**Figure 6:**
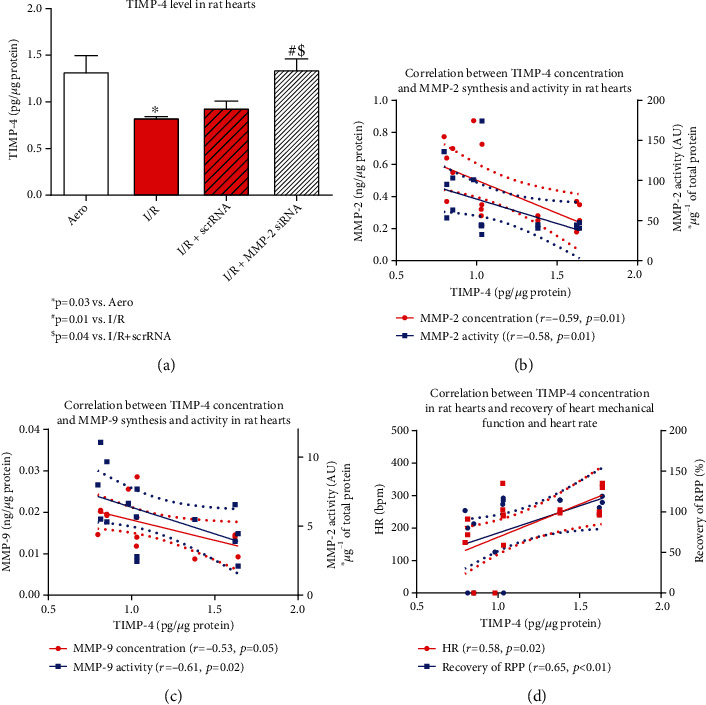
TIMP-4 level in rat hearts (a). Correlation between TIMP-4 level and MMP-2 (b) and MMP-9 (c) synthesis/activity in rat hearts. Correlation between TIMP-4 level in rat hearts and HR/RPP (d). Straight line: standard curve; dotted line: 95% confidence band. Aero: aerobic control group; I/R: ischemia/reperfusion; MMP-2: matrix metalloproteinase 2; TIMP-4: endogenous tissue inhibitor of matrix metalloproteinase 4.

TIMP-4 was significantly decreased in the I/R hearts (*p* = 0.03), confirming the disruption of its function due to ischemia/reperfusion. Administration of siRNA silencing MMP-2/MMP-9 restored the concentration of TIMP-4 to the level observed in aerobically perfused hearts (*p* = 0.01 vs. I/R,*p* = 0.04 vs. I/R+scrRNA) (Figure 6(a)). Moreover, negative correlations between TIMP-4 level and MMP-2/MMP-9 synthesis and activity in heart tissue (MMP-2: *p* = 0.01, *r* = −0.59; *p* = 0.01, *r* = −0.58, respectively; MMP-9: *p* = 0.05, *r* = −0.53; *p* = 0.02, *r* = −0.61, respectively) were observed (Figures 6(b) and 6(c)). We also revealed a positive correlation of TIMP-4 level with HR and recovery of heart mechanical function (*p* = 0.02, *r* = 0.58; *p* < 0.01, *r* = 0.65, respectively) (Figure 6(d)). This confirmed that MMPs-TIMP-4 balance in heart was disturbed due to I/R injury, and it is reflected in the deterioration of hemodynamic parameters. However, MMP-2 siRNA restored proper balance.

## 4. Discussion

It was previously found that pharmacological inhibition of MMP-2 may reduce injury caused by ischemia and reperfusion [4, 5, 12, 19, 29]. Our previous study on rat hearts subjected to I/R showed substantially increased MMP-2 activity; however, administration of doxycycline, L-NAME, and ML-7 (MMP-2, nitric oxide synthase (NOS), and myosin light chain kinase (MLCK) inhibitors) to I/R hearts resulted in the normalization of MMP-2 activity and protection of heart function [30]. It has also been reported that the use of doxycycline or o-phenanthroline alone may slightly reduce infarct size, significantly weaken myocardial remodeling, and improve the reversible mechanical dysfunction of heart damaged during I/R [31, 32].

Pathophysiology underlying heart IRI is still intriguing the scientist and clinicians trying to determine the most effective treatment method. A great diversity of therapeutic approaches has already been proposed, but there is still no agreement on this matter. Given the importance of assessing and controlling the pathological processes in heart tissue, it seems crucial to focus on finding new strategies to reduce I/R effects. For several years, the method of gene silencing with the use of siRNA is increasingly applied in the treatment of vascular diseases [23]. In the current study, we investigated how the administration of MMP-2 siRNA affected hearts subjected to ischemia/reperfusion injury. We analyzed the sequence of MMP-2 siRNA used in the experiment and determined that it is able to attach to MMP-2 but also to MMP-9 mRNA; thus, we explored the effect of siRNA also on MMP-9 protein. We revealed that *MMP-2* as well as *MMP-9* gene expressions were inhibited after siRNA administration to the heart. We also showed that both MMP-2 and MMP-9 synthesis and activity were reduced after the administration of siRNA. Lin et al. (2014) have already proved that administration of MMP-2 siRNA to isolated myocytes reduced synthesis and activity of MMP-2. This change was associated with an improvement in myocyte contractility [8]. MMP-2 siRNA was also used as a potential therapeutic strategy against vascular diseases [23]. Hlawaty et al. injected MMP-2 siRNA into rabbit vascular smooth muscle cells (VSMC), which resulted in inhibition of MMP-2 expression and activity in VSMC [23].

An increase of MMP-2 and MMP-9 protein synthesis and activity in I/R hearts was associated with decreased TIMP-4 level, probably due to its expenditure since it is endogenous MMPs inhibitor. TIMPs bind noncovalently to active MMPs in a molar ratio of 1 : 1 [19]. Inhibition of the enzyme proteolytic activity is then possible due to the ability of TIMP to interact with the zinc-binding site within the catalytic domain of active MMPs [5, 18, 19]. Except for TIMP-1, it is known that all TIMPs are capable of inhibiting all MMPs [10, 19]. Therefore, TIMP-4, which is most abundant in the heart, should inhibit the activity of MMP-2 and MMP-9 under physiological conditions [2, 3, 5]. The imbalance between MMP and TIMP, observed in our study, may contribute to cardiac injury after I/R [15]. TIMP-4 is strongly expressed in the cardiovascular system under physiological conditions, and numerous studies showed a relative reduction of TIMP-4 expression level after infarction [3, 5, 18, 33, 34]. Our results showed that TIMP-4 level was normalized to the level of aerobic control after treatment with MMPs siRNA, accompanied by decreased activity of MMPs. This suggests that the balance between MMPs and TIMP-4 may be restored, leading to cardioprotection. To confirm that, we showed a negative correlation between MMP-2 and MMP-9 synthesis and activity with TIMP-4 level in rat hearts. Importantly, we also observed that TIMP-4 level positively correlated with recovery of the heart mechanical function and heart rate, confirming that it is involved in restoring proper heart function.

ROS contribute to I/R injury through MMP-2 activation and by posttranslational modifications of heart contractile proteins: phosphorylation, nitration, and nitrosylation. Posttranslational modifications of contractile proteins intensify their degradation by MMP-2 [30, 35–37], and it is well established that this process contributes significantly to systolic dysfunction [30, 36]. LDH, which was used as a marker of cell death due to IRI, substantially increased in coronary effluents during I/R indicating cell damage, while its release was inhibited after siRNA treatment. We also showed that both TnI and MLC1 proteins were released in substantially greater amounts into coronary effluents from I/R hearts compared to aerobic control. To confirm the association between heart damage and contractile proteins degradation due to I/R, a positive correlations between LDH activity in coronary effluents and TnI and MLC1 release into extracellular space were observed. MMP siRNA inhibited the release of TnI and MLC1 from hearts confirming decreased proteolysis of these proteins. To support this thesis, we showed that TnI as well as MLC1 release into extracellular space positively correlated with MMP-2 activity in the heart tissue. Moreover, TnI release positively correlated with MMP-9 synthesis, which suggest that this protein was also associated with degradation of contractile proteins during IRI.

siRNA transfection resulted in MMP-2 and MMP-9 inhibition, and then, reduction of their synthesis protected MLC1, MLC2, and hence heart rate against the effects of I/R [8]. Hughes and Schulz reported that cardiac TnI levels were reduced in isolated hearts of rats subjected to I/R and the use of MMP-2 inhibitors such as okadaic acid prevented TnI loss and reduced the severity of post-I/R systolic dysfunction [19]. Moreover, we proved in our previous study that the level of MLC1, which is a specific marker of heart damage, was reduced in I/R heart tissue by approximately 30%, while the treatment of hearts with a mixture of MMP-2, MLCK and NOS inhibitors prevented such decrease [37]. It was also observed that the activity of MMP-2 was normalized after the use of inhibitors mixture, and a strong negative correlation between MLC1 and MMP-2 was observed in hearts subjected to I/R [37]. In the current research, we found a corresponding positive correlation between MLC1 release into coronary effluents and MMP-2 activity in the hearts. Kandasamy et al. and Ferb-Bober et al. also reported that inhibition of MMP-2 activity in isolated rat hearts using doxycycline or o-phenanthroline prevented I/R damage-induced degradation of TnI and MLC1 and improved the mechanical function of the heart [12, 15]. MMP-9 protein was also linked to post-I/R cardiac remodeling, direct impairment of the contractile apparatus, and cell apoptosis [38, 39]. MMP-9 deficiency was also found to protect against cardiac rupture [39]. Manginas et al. showed that patients with acute coronary syndromes had increased level of MMP-9 which correlated with increased serum TnI and other inflammatory mediators such as IL-6 or CRP [40]. It is suggested that TnI was released into serum due to inflammation [40]. TnI constitutes a key regulator of the cardiomyocyte contractile mechanism and interacts with actin to prevent its binding with myosin [31]. Muscle contraction occurs when Ca^2+^ induces conformational changes in the troponin protein, leading to TnI and tropomyosin dislocation, which in turn allows myosin to interact with actin (cross-bridge formation) [31, 32]. MLC1 plays an important role in the contraction of the heart muscle by stabilizing the myosin neck area and thus influences the formation of cross-bridges between myosin and actin [41]. Increased degradation of TnI and MLC1 is a common feature for myocardial stunning [12].

Going further, increased activity of MMP-2 and MMP-9 during heart I/R injury affected heart mechanical function, which was confirmed by their negative correlation. Since tissue injury and release of contractile proteins were decreased due to MMP-2 siRNA administration, heart hemodynamic parameters returned to the baseline (aerobic control). Similar results were also observed in our previous study where the mixture acting on MMP-2, nitrates, and MLCK was used [30]. Fert-Bober et al. and Gomori et al. also reported that HR, RPP, and CF were decreased in I/R hearts and an improvement in these parameters was visible after the use of doxycycline and/or o-phenanthroline [12, 32].

## 5. Conclusions

This study revealed that siRNA homologous to MMP-2 and MMP-9 conserved region of the gene has a potential of cardioprotective factor in hearts subjected to ischemia/reperfusion injury through the reduction of MMP-2 and MMP-9 levels and activities in the heart and restoration of the balance between those proteins and their endogenous inhibitor—TIMP-4. On this basis, reduced degradation of contractile proteins as well as reduced heart damage was observed. An improvement of heart mechanical function confirmed that siRNA may serve as a standard manner for acute coronary syndromes in the future. We believe that the results obtained in this study may complement the information on the mechanisms of myocardial damage as a result of I/R at the molecular level and fill in the lack of knowledge about the intracellular regulation of MMP-2 activity. This, in turn, may form the basis for the development of a new and effective therapy for heart damage resulting from coronary revascularization. The results obtained in this project may also provide fundamental information on the effectiveness of therapy with a selective MMP-2/MMP-9 inhibitor on the improvement of cardiac hemodynamic functions.

## Figures and Tables

**Table 1:** An influence of MMP-2 siRNA on heart rate, LVDP, coronary flow, and recovery of heart functions of isolated rat hearts.

Parameter	Groups					*p* value^d^
	Aero	Aero+MMP-2 siRNA and siPORT	I/R	I/R+scr RNA	I/R+MMP-2 siRNA and siPORT	
HR (bpm)^a^	267.2 ± 8.6	264.5 ± 0.5	127.6 ± 45.4^∗^	106.6 ± 44.4^∗^	284.0 ± 5.4^#$^	<0.01
LVDP (mmHg)^a^	53.9 ± 3.5	75.5 ± 10.5	29.7 ± 10.8^∗^	18.8 ± 7.9^∗^	71.3 ± 9.3^#$^	<0.01
CF (mL/min)^b^	13.5 ± 0.8	14.7 ± 1.8	8.3 ± 1.9^∗^	5.2 ± 2.1^∗^	12.6 ± 0.7^#$^	<0.01
Recovery of RPP (%)^c^	92.2 ± 2.7	105.0 ± 17.0	54.2 ± 10.8^∗^	28.8 ± 13.6^∗^	116.6 ± 9.3^#$^	<0.001

Mean ± SEM. ^a^After I/R (77^th^ minute of the experiment). ^b^After ischemia (47^th^ minute: first minute of reperfusion). ^c^Difference between RPP in 25^th^ and 77^th^ minutes of experiment. ^d^ANOVA test; ^∗^*p* < 0.05 vs. aerobic; ^#^*p* < 0.05 vs. I/R, $*p* < 0.05 vs. I/R+scrRNA. Aero: aerobic control group; CF: coronary flow; HR: heart rate; I/R: ischemia-reperfusion group; LVDP: left ventricular developed pressure; MMP-2: matrix metalloproteinase 2; RPP: rate pressure product.

## Data Availability

The datasets used and/or analyzed during the current study are available from the corresponding author on reasonable request.

## References

[1] Kargiotis O., Chetty C., Gondi C. S. (2008). Adenovirus-mediated transfer of siRNA against MMP-2 mRNA results in impaired invasion and tumor-induced angiogenesis, induces apoptosis _in vitro_ and inhibits tumor growth _in vivo_ in glioblastoma. *Oncogene*.

[2] Hojo Y., Ikeda U., Ueno S., Arakawa H., Shimada K. (2001). Expression of matrix metalloproteinases in patients with acute myocardial infarction. *Japanese Circulation Journal*.

[3] Zavadzkas J. A., Stroud R. E., Bouges S. (2014). Targeted overexpression of tissue inhibitor of matrix metalloproteinase-4 modifies post-myocardial infarction remodeling in mice. *Circulation Research*.

[4] Matsumura S., Iwanaga S., Mochizuki S., Okamoto H., Ogawa S., Okada Y. (2005). Targeted deletion or pharmacological inhibition of MMP-2 prevents cardiac rupture after myocardial infarction in mice. *The Journal of Clinical Investigation*.

[5] Creemers E. E. J. M., Cleutjens J. P. M., Smits J. F. M., Daemen M. J. A. P. (2001). Matrix metalloproteinase inhibition after myocardial infarction. *Circulation Research*.

[6] DeCoux A., Lindsey M. L., Villarreal F., Garcia R. A., Schulz R. (2014). Myocardial matrix metalloproteinase-2: inside out and upside down. *Journal of Molecular and Cellular Cardiology*.

[7] Katus H. A., Remppis A., Neumann F. J. (1991). Diagnostic efficiency of troponin T measurements in acute myocardial infarction. *Circulation*.

[8] Lin H.-B., Cadete V. J. J., Sra B. (2014). Inhibition of MMP-2 expression with SiRNA increases baseline cardiomyocyte contractility and protects against simulated ischemic reperfusion injury. *BioMed Research International*.

[9] Wang L. L., Chung J. J., Li E. C., Uman S., Atluri P., Burdick J. A. (2018). Injectable and protease-degradable hydrogel for SiRNA sequestration and triggered delivery to the heart. *Journal of Controlled Release*.

[10] Wilson E. M., Gunasinghe H. R., Coker M. L. (2002). Plasma matrix metalloproteinase and inhibitor profiles in patients with heart failure. *Journal of Cardiac Failure*.

[11] Yarbrough W. M., Mukherjee R., Brinsa T. A. (2003). Matrix metalloproteinase inhibition modifies left ventricular remodeling after myocardial infarction in pigs. *The Journal of Thoracic and Cardiovascular Surgery*.

[12] Fert-Bober J., Leon H., Sawicka J. (2008). Inhibiting matrix metalloproteinase-2 reduces protein release into coronary effluent from isolated rat hearts during ischemia-reperfusion. *Basic Research in Cardiology*.

[13] Fan Z., Fu M., Xu Z. (2017). Sustained release of a peptide-based matrix metalloproteinase-2 inhibitor to attenuate adverse cardiac remodeling and improve cardiac function following myocardial infarction. *Biomacromolecules*.

[14] Baghirova S., Hughes B. G., Poirier M., Kondo M. Y., Schulz R. (2016). Nuclear matrix metalloproteinase-2 in the cardiomyocyte and the ischemic- reperfused heart. *Journal of Molecular and Cellular Cardiology*.

[15] Kandasamy A. D., Chow A. K., Ali M. A. M., Schulz R. (2010). Matrix metalloproteinase-2 and myocardial oxidative stress injury: beyond the matrix. *Cardiovascular Research*.

[16] Tong W., Xiong F., Li Y., Zhang L. (2013). Hypoxia inhibits cardiomyocyte proliferation in fetal rat hearts via upregulating TIMP-4. *American Journal of Physiology-Regulatory, Integrative and Comparative Physiology*.

[17] Koskivirta I., Rahkonen O., Mäyränpää M. (2006). Tissue inhibitor of metalloproteinases 4 (TIMP4) is involved in inflammatory processes of human cardiovascular pathology. *Histochemistry and Cell Biology*.

[18] Webb C. S., Bonnema D. D., Ahmed S. H. (2006). Specific temporal profile of matrix metalloproteinase release occurs in patients after myocardial infarction: relation to left ventricular remodeling. *Circulation*.

[19] Hughes B. G., Schulz R. (2014). Targeting MMP-2 to treat ischemic heart injury. *Basic Research in Cardiology*.

[20] Lindsey M. L. (2004). MMP induction and inhibition in myocardial infarction. *Heart Failure Reviews*.

[21] Sun Y., Liu M., Yang B., Li B., Lu J. (2008). Role of SiRNA silencing of MMP-2 gene on invasion and growth of laryngeal squamous cell carcinoma. *European Archives of Oto-Rhino-Laryngology*.

[22] Maheshwari R., Tekade M., Sharma P. A., Tekade R. K. (2015). Nanocarriers assisted siRNA gene therapy for the management of cardiovascular disorders. *Current Pharmaceutical Design*.

[23] Hlawaty H., San Juan A., Jacob M.-P., Vranckx R., Letourneur D., Feldman L. J. (2007). Inhibition of MMP-2 gene expression with small interfering RNA in rabbit vascular smooth muscle cells. *American Journal of Physiology-Heart and Circulatory Physiology*.

[24] Lindsey M. L., Bolli R., Canty J. M. (2018). Guidelines for experimental models of myocardial ischemia and infarction. *American Journal of Physiology-Heart and Circulatory Physiology*.

[25] Bøtker H. E., Hausenloy D., Andreadou I. (2018). Practical guidelines for rigor and reproducibility in preclinical and clinical studies on cardioprotection. *Basic Research in Cardiology*.

[26] Krzywonos-Zawadzka A., Franczak A., Olejnik A. (2019). Cardioprotective effect of MMP-2-inhibitor-NO-donor hybrid against ischaemia/reperfusion injury. *Journal of Cellular and Molecular Medicine*.

[27] Biały D., Wawrzyńska M., Bil-Lula I. (2018). Low frequency electromagnetic field decreases ischemia-reperfusion injury of human cardiomyocytes and supports their metabolic function. *Experimental Biology and Medicine (Maywood, N.J.)*.

[28] Heussen C., Dowdle E. B. (1980). Electrophoretic analysis of plasminogen activators in polyacrylamide gels containing sodium dodecyl sulfate and copolymerized substrates. *Analytical Biochemistry*.

[29] Kelly D., Cockerill G., Ng L. L. (2007). Plasma matrix metalloproteinase-9 and left ventricular remodelling after acute myocardial infarction in man: a prospective cohort study. *European Heart Journal*.

[30] Krzywonos-Zawadzka A., Franczak A., Sawicki G., Bil-Lula I. (2020). Mixture of MMP-2, MLC, and NOS inhibitors affects NO metabolism and protects heart from cardiac I/R injury. *Cardiology Research and Practice*.

[31] Dejonckheere E., Vandenbroucke R. E., Libert C. (2011). Matrix metalloproteinases as drug targets in ischemia/reperfusion injury. *Drug Discovery Today*.

[32] Gömöri K., Szabados T., Kenyeres É. (2020). Cardioprotective effect of novel matrix metalloproteinase inhibitors. *IJMS*.

[33] Rouet-Benzineb P., Buhler J.-M., Dreyfus P. (1999). Altered balance between matrix gelatinases (MMP-2 and MMP-9) and their tissue inhibitors in human dilated cardiomyopathy: potential role of MMP-9 in myosin-heavy chain degradation. *European Journal of Heart Failure*.

[34] Schulze C. J., Wang W., Suarez-Pinzon W. L., Sawicka J., Sawicki G., Schulz R. (2003). Imbalance between tissue inhibitor of metalloproteinase-4 and matrix metalloproteinases during acute myoctardial ischemia-reperfusion injury. *Circulation*.

[35] Horowitz J. D., Chong C.-R. (2020). Matrix metalloproteinase-2 activation: critical to myocardial contractile dysfunction following ischaemia–reperfusion. *Cardiovascular Research*.

[36] Cadete V. J. J., Sawicka J., Bekar L. K., Sawicki G. (2013). Combined subthreshold dose inhibition of myosin light chain phosphorylation and MMP-2 activity provides cardioprotection from ischaemic/reperfusion injury in isolated rat heart. *British Journal of Pharmacology*.

[37] Krzywonos Zawadzka A., Woźniak M., Sawicki G., Bil-Lula I. (2020). A drug cocktail for protecting against ischemia-reperfusion injury. *Frontiers in Bioscience*.

[38] Halade G. V., Jin Y.-F., Lindsey M. L. (2013). Matrix metalloproteinase (MMP)-9: a proximal biomarker for cardiac remodeling and a distal biomarker for inflammation. *Pharmacology & Therapeutics*.

[39] Reinhardt D., Sigusch H. H., Hensse J., Tyagi S. C., Körfer R., Figulla H. R. (2002). Cardiac remodelling in end stage heart failure: upregulation of matrix metalloproteinase (MMP) irrespective of the underlying disease, and evidence for a direct inhibitory effect of ACE inhibitors on MMP. *Heart*.

[40] Manginas A., Bei E., Chaidaroglou A. (2005). Peripheral levels of matrix metalloproteinase-9, interleukin-6, and C-reactive protein are elevated in patients with acute coronary syndromes: correlations with serum troponin I. *Clinical Cardiology*.

[41] Banaszkiewicz M., Krzywonos-Zawadzka A., Olejnik A., Bil-Lula I. (2020). Tissue expression of atrial and ventricular myosin light chains in the mechanism of adaptation to oxidative stress. *IJMS*.

